# Nimesulide inhibited the growth of hypopharyngeal carcinoma cells via suppressing Survivin expression

**DOI:** 10.1186/1758-3284-4-7

**Published:** 2012-03-27

**Authors:** Tian Jia-Jun, Lu Su-Mei, Yu Liang, Ma Ju-Ke, Mu Ya-Kui, Wang Hai-Bo, Xu Wei

**Affiliations:** 1 Department of Otolaryngology-Head and Neck Surgery, Provincial Hospital affiliated to Shandong University, Jinan 250021, PR, China; 2 Institute of Eye & Otolaryngology, Shandong Clinic Research Institute, Jinan 250021, PR, China

**Keywords:** Hypopharyngeal carcinoma, Cell growth, Nimesulide, COX-2

## Abstract

**Background:**

The objective of this study was to evaluate the efficacy of Nimesulide, a selective cyclooxygenase-2 (COX-2) inhibitor, on the growth of hypopharyngeal carcinoma cells (FaDu) in vitro, and investigate its potential mechanism.

**Methods:**

After FaDu cells were treated with graded concentrations of Nimesulide for divergent time, sensitivity of cells to drug treatment was analyzed by MTT assay. Morphological changes of FaDu cells in the presence of Nimesulide were observed by acridine orange cytochemistry staining. Proliferating cells were detected using the 5-Bromo-2'-deoxy-uridine (BrdU) incorporation assay. Following cells were subjected to Nimesulide (500 μmol/l) for 6 h, 12 h and 24 h, the percentage of apoptosis was examined by flow cytometry. We detected COX-2 and Survivin expression change by RT-PCR and Western blot, and analyzed the correlation of them with the growth of FaDu cells. Additionally, we also analyzed Caspase-3, Bcl-2 and Bax expressions as markers to investigate the related pathway of Nimesulide-indued apoptosis.

**Results:**

Compared with the control group, the viabilities rates were decreased by Nimesulide in time- and dose-dependent manners, typical morphological changes of apoptotic cells were observed in the Nimesulide-treatment groups, Nimesulide could suppress the proliferation of FaDu cells significantly. The percentage of apoptosis in FaDu cells were markedly increased after Nimesulide-treatment for 6 h, 12 h and 24 h. Nimesulide down-regulated the Survivin and COX-2 expressions at mRNA and protein levels in FaDu cells. Additional analyses indicated that Bcl-2 expression was significantly decreased and the expressions of Caspase-3 as well as Bax were increased at both mRNA and protein levels.

**Conclusions:**

Based on the induction of apoptosis and suppression of proliferation, Nimesulide could inhibit the growth of FaDu cells. Furthermore, the suppression of Survivin expression may play an important role in Nimesulide-induced growth inhibition. Nimesulide could act as an effective therapeutic agent for hypopharyngeal carcinoma therapy.

## Background

COX-2, a rate-limiting enzyme involved in conversion of arachidonic acid to prostaglandins (PGs), has been reported over-expression in a wide range of human malignant tumors. More recently, it has been demonstrated that COX-2 could contribute to carcinogenesis, prompt tumor growth and progression, and closely associate with higher risk of metastasis as well as poor survival [1-3]. Previous studies have suggested that the inhibition of COX-2 has anticancer effects in many kinds of cancers. Suppressing COX-2 expression has effects on inhibition of carcinogenesis, and further may suppress the invasion of advanced cancer [4,5]. Up to now, although many studies have demonstrated that the selective COX-2 inhibitors were useful agents on the cancer therapy, the exact mechanisms by which the COX-2 played anti-cancer effect remain unclear. However, the administration of selective COX-2 inhibitors has come into research focus in the development of new anti-cancer agents.

Survivin, a member of inhibitor of apoptosis protein (IAP) family, is highly expressed in most human cancers but not in normal tissues, and the up-regulation of Survivin expression generally means predictive of tumor progression and poor prognosis. Its function in mitosis is to preserve the mitotic apparatus and to allow normal mitotic progression. Survivin is involved in the process of apoptosis and its anti-apoptotic function is executed via preventing Caspases activation [6,7]. A report from Mehar et al. showed that COX-2 over-expression increased levels of Survivin [8].

Hypopharyngeal carcinoma is common malignancy in the head and neck region. Recently studies have demonstrated that COX-2 over-expression was detected in hypopharyngeal carcinomas [9,10]. However, there was no data available regarding the anticancer effects of selective COX-2 inhibitors on hypopharyngeal carcinomas. In present study, we firstly treated hypopharyngeal carcinoma cell line with Nimesulide, a selective COX-2 inhibitor, and evaluated its effects on the growth of FaDu cells. Furthermore, we also detected the expression of Survivin to investigate its role in Nimesulide-induced growth inhibition.

## Methods

### Cell culture and drug treatment

FaDu cells was purchased from Shanghai Biosis Biotechnology Co.Ltd.. FaDu (approximately 15 × 10^4 ^cells/ml) was seeded in culture flasks and incubated in DMEM supplemented with 10% FBS at 37°C in the presence of 5% CO_2 _in a humidified incubator. Nimesulide were purchased from Sigma. Concentrated drug stocks were diluted in DMEM before administration to cells. Subsequently, Nimesulide were added to the medium (each at final concentration of 500 μ mol/l), and according to the result of our pre-experiment, the effect of necrosis is higher than apoptosis, so FaDu cells were harvested after 6 h, 12 h of incubation, only the cells in flow cytometry were incubated for 24 h.

### MTT assay

MTT was performed to evaluate sensitivity of FaDu cells to Nimesulide treatment. Briefly, FaDu cells were respectively plated in a 96-well plate and further incubated for 24 h. Then, the medium was removed and cells were exposed to fresh medium containing various combinations of Nimesulide (10 μM to1000 μM) for 12 h, 24 h, and 48 h. 20 μl MTT (5 g/l) was added to each well and cultured for additional 4 h. Subsequently, the supernatant was removed, 100 μl DMSO was added into each well and shaken for another 20 min until the crystals were dissolved. The absorption (A) was measured at 570 nm. Controls consisted of untreated cells. Three independent experiments were performed for each group.

### Morphological observation

Morphological changes of FaDu cells in response to Nimesulide treatment were evaluated by staining cells with acridine orange. Briefly, after 24 h of incubation, the medium was removed and refilled with new serum-free medium containing Nimesulide (500 μ mol/l) for 6 h and 12 h incubation. Then the cells were stained by 0.01% acridine orange for 5 min, the morphological changes were examined by fluorescence microscope. Cells with typical features of nuclear fragmentation and/or marked condensation of chromatin with reduced nuclear size were interpreted as apoptotic cells.

### BrdU assay

The effect of Nimesulide on proliferation of FaDu cells was detected by the BrdU incorporation assay. 15 × 10^4 ^cells/ml FaDu cells was seeded onto a cover glass in 24-well plates. FaDu cells were treated with or without Nimesulide (500 μmol/l) for 12 h, respectively. As described in manufacturer's manual (Roche Applied Science), BrdU labeling medium was added to the cells for 4 h in 37°C incubator. After washing the cells with phosphate buffered solution (PBS) three times, cells was incubated with anti-BrdU antibody in incubation buffer overnight at 4°C, followed by incubation with anti-mouse antibody for 30 min at 37°C. 4', 6-diamidino-2-phenylindole dihydrochloride (DAPI, Sigma-Aldrich) (2 μg/ml) was used as nuclear staining for counterstaining. Images were captured with a fluorescence microscope, and the percentage of BrdU-labeled cells was calculated.

### Flow cytometry analysis

Cell apoptotic progression was assessed by flow cytometry using Annexin V-FITC and propidium iodide (PI). Cells were harvested by trypsinized, washed with phosphate-buffered saline (PBS). After washing, cells (1 × 10^6^/ml) were resuspended in Binding Buffer (200 μl) containing Annexin V-FITC (5 μl, 20 μg/ml) and PI (10 μl, 20 μg/ml) for at least 15 min at room temperature, and then added Binding Buffer (300 μl) before analysis with a SystemII(ver3.0) (Beckmand).

### RNA extraction and RT-PCR

RNA was extracted using TRIzol (Invitrogen) from cultured cells after being incubated with Nimesulide (500 μmol/l) for 6 h and 12 h respectively. RNA was also extracted from control group. The RT-PCR was performed following the instruction of TaKaRa TaqTM to determine the expession of Caspase-3, Bcl-2, Bax, Survivin and COX-2. β-actin was used as standard. The primers for the respective genes were designed as follows:

Bax: F: 5'GTGCACCAAGGTGCCGGAAC3';

R: 5'TCAGCCCATCTTCTTCCAGA3';

Bcl-2: F: 5'CGACGACTTCTCCCGCCGCTACCGC3';

R: 5'CCGCATGCTGGGGCCGTACAGTTCC3';

Caspase-3: F: 5' AAATGGACCTGTTGACCTGA3';

R: 5' GCACAAAGCGACTGGATG3'

Survivin: F: 5'CAGATTTGAATCGCGGGACCC3'

R: 5'CCAAGTCTGGCTCGTTCTCAG3'

COX-2: 5' CAGGGTTGCTGGTGGTAGGA 3';

5' GAACACTGAATGAAGTAAAGGGACA 3';

β-actin: F: 5' GTGGGGCGCCCCAGGCACCA 3';

R: 5' GTGGGGCGCCCCAGGCACCA 3'

PCR products were electrophoresed on a 1% agarose gel. The gel was then visualized and photographed under UV light. The mRNA expressions were determined by comparing with β-actin. All experiments were done in triplicate.

### Protein extraction and western blot analysis

After protein extraction and determining the protein concentration of the supernatant by Bradford method, protein was separated by electrophoresis in 12.5% sodium dodecyl sulfate polyacrylamide gel electrophoresis (SDS-PAGE) gels according to their molecular weight. After incubation with primary antibodies (Rabbit anti-Human COX-2, 1:300; Survivin, 1:1000; Caspase-3, Bcl-2 and Bax, 1:200; Mouse anti-human β-actin, 1:2000), and then, incubated with peroxidase-linked secondary antibody. Reactive bands were detected using ECL chemiluminescence reagent (Amersham Biosciences). The β-actin, COX-2, Survivin, Caspase-3, Bcl-2 and Bax bands were visualized at apparent molecular weights of 43 kDa, 72 kDa, 16 kDa, 32 kDa, 26 kDa and 23 kDa, respectively. Relative OD value ratio was calculated with NIH software Image J by comparing to β-actin from triplicate experiments.

### Statistical analysis

Correlation analysis between expressions of target genes in clinical tumor samples was done using the Student's *t *test. For statistical evaluation of cell culture experiments, mean values and SD of the triplicate experiments were calculated. Gene expression levels under treatment with Nimesulide were compared with the respective expression levels without drug treatment using Anova test. SPSS 13. 0 statistical software (SPSS Inc) was used. P values smaller 0.05 were considered as statistically significant.

## Results

### Nimesuide suppressed the viability of FaDu cells

**Figure 1:**
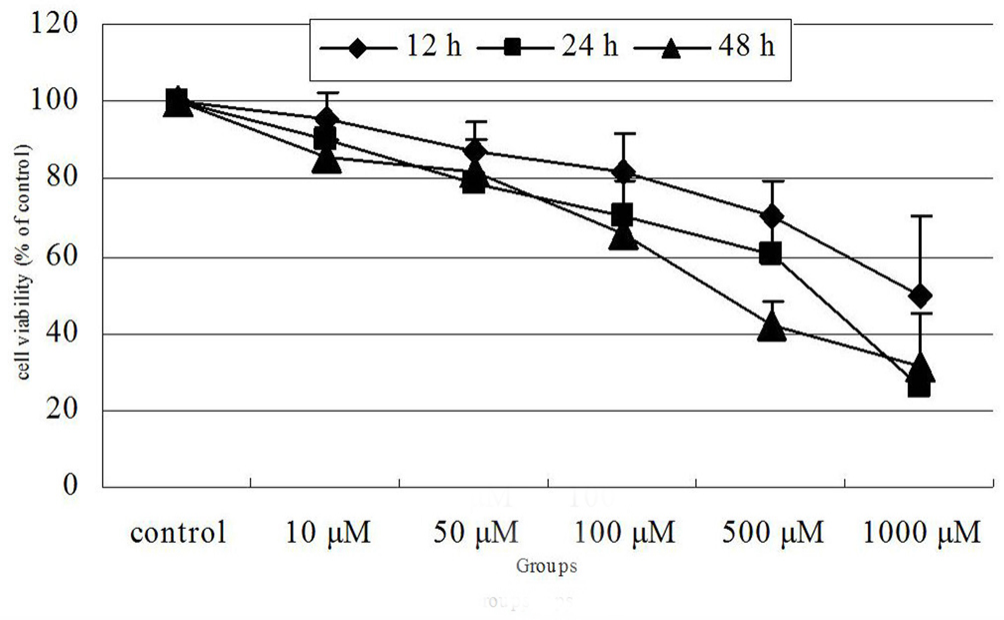
**Effects of Nimesulide on viability of FaDu cells**. The anti-proliferating effects of Nimesulide were evaluated by MTT assay. Cells were treated with 0-1000 μM Nimesulide and the percentage of growth inhibition was determined after 12 ~ 48 h of treatment. FaDu cells had significant time- and dose-dependent growth inhibition due to Nimesulide.

As shown in Figure 1, the relative viability of cells was suppressed after incubation with Nimesulide. Taken control as 100%, the relative viabilities of FaDu cells exposure to Nimesulide at 10 μmol/l, 50 μmol/l, 100 μmol/l, 500 μmol/l, 1000 μmol/l for 24 h were (89.90 ± 7.83)%, (78.80 ± 6.69)%, (70.57 ± 8.52)%, (60.10 ± 7.26)% and (26.12 ± 11.7)%, respectively (*P < 0.05*). Taken the viability of FaDu cells exposure to Nimesulide (500 μmol/l) at 0 h as 100%, the relative viabilities of FaDu cells at 12 h, 24 h and 48 h were (70.6 ± 8.81)%, (60.10 ± 7.26)% and (42.2 ± 5.59)% (*P < 0.05*).

### Effects of nimesulide on morphological changes in FaDu cells

**Figure 2:**
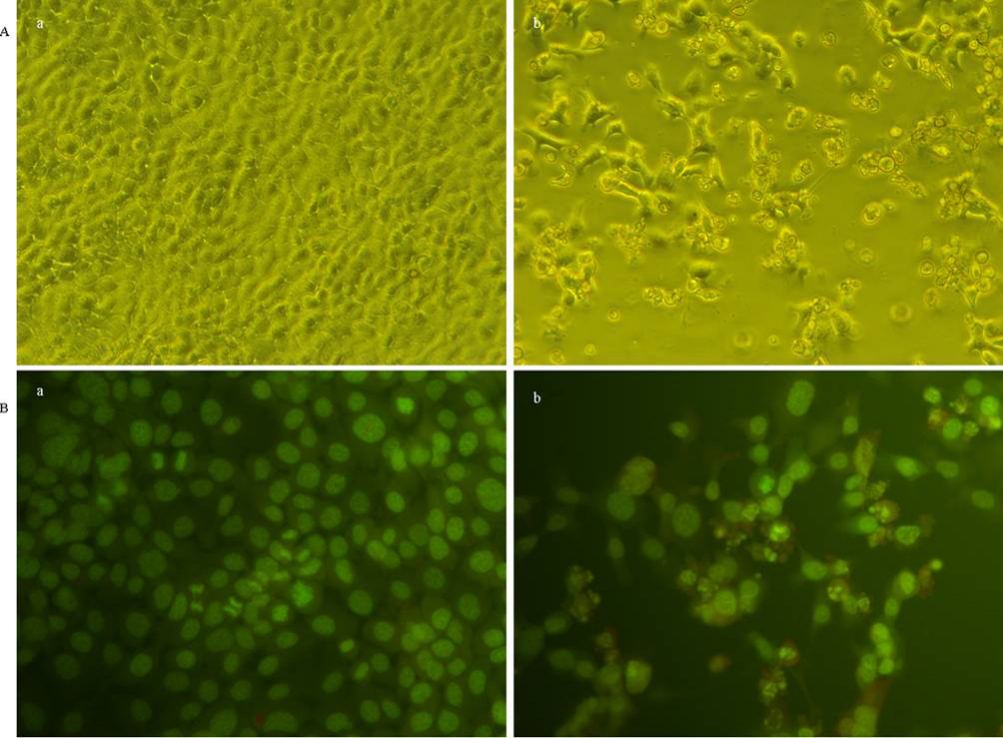
**Morphological changes of FaDu cells before and after Nimesulide-treatment (500 μmol/l, 24 h)**. Cells were stained with acridine orange and detected by fluorescence microscopy (×200). A: The number of survival cells in Nimesulide treated group was much fewer than that of control. B: Morphological changes and apoptotic bodies were detected obviously in Nimesulide group.

Morphological changes of FaDu cells were examined by inverted phase contrast microscope and acridine orange cytochemistry staining at 6 h or 12 h after treated with Nimesulide (500 μmol/l). As shown in Figure 2, compared to control, the number of cells was significantly decresed after Nimesulide treatment. The cells without Nimesulide treatment showed polygonal shape, but, the cells treated with Nimesulide for 12 h became rounded and had cytoplasmic contraction and chromatin condensation. Apoptotic bodies, as the main morphological characteristic of apoptosis, were detected obviously in cells treated with Nimesulide.

### BrdU assay on proliferating of FaDu cells

**Figure 3:**
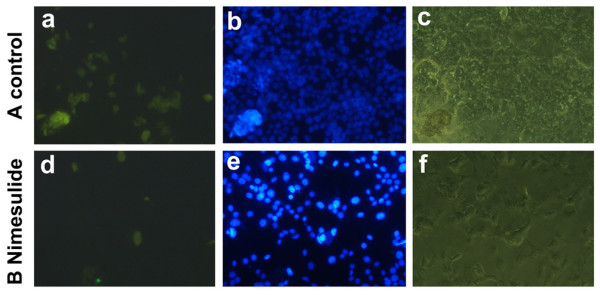
**Inhibition of cell proliferation was detected by BrdU incorporation assay after treatment with Nimesulide (500 μmol/l)**. A: control group; B: treated with Nimesulide (500 μmol/l). a/d: The BrdU stained cells by immunofluorescence was detected by fluorescence microscopy (×200); b/e: nuclear stained by DAPI was detected by fluorescence microscopy (×200), c/f: Direct detected by reserved microscopy (×200).

The number of the BrdU positive cells detected in control group was more than that of Nimesulide treated group (Figure 3) (*P < 0.05*). Compared to control, the percentage of BrdU positive cells was significantly decreased to 30% in cells with Nimesulide (500 μmol/l, 12 h). These results were consistent with previous data showing that Nimesulide helps to decrease the viability of FaDu cells.

### Flow cytometry (FCM) analysis

**Figure 4:**
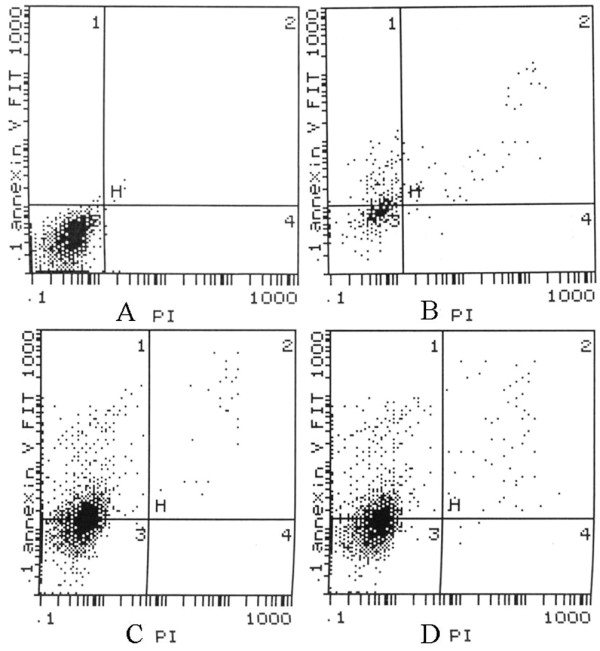
**Apoptotic rates of FaDu cells treated with Nimesulide were detected using FCM**. The percentage of apoptosis cells was markedly increased in the present of Nimesulide. × -axis represents the density of propidium iodide (PI), Y- axis represents the density of Annexin V-FITC. A: control; B: 6 h, C: 12 h; D: 24 h.

Flow cytometric analysis (Figure 4) showed that the apoptotic rates at 6 h, 12 h, or 24 h after the administration of Nimesulide (500 μmol/l) were (32.4 ± 6.1)% and (45.87 ± 3.19)%, respectively, whereas, the control was (4.17 ± 4.20)% (*P < 0.05*). After cells were incubated for 24 h, the effect of necrosis is higher than apoptosis; the apoptotic rate was (44.57 ± 4.10)%.

### The expression of survivin and COX-2 in FaDu cells after nimesulide treatment

**Figure 5:**
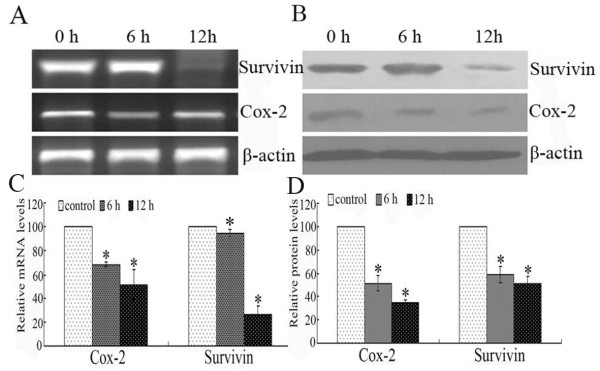
**Alterations of Survivin and COX-2 expressions in FaDu cells**. A: RT-PCR analysis. B: Western blot detection. The optical densities (OD) value of mRNA (C) and proteins (D) in each lane using NIH software Image J system were demonstrated in different times treated with Nimesulide. Each data point in the figure represents the mean ± SEM of three separate experiments.

As shown in Figure 5, compared with control, the mRNA and protein levels of both Survivin and COX-2 were all suppressed after treatment with Nimesulide for 6 h and 12 h. Concretely, taken control as 100%, after FaDu were treated with Nimesulide for 6 h and 12 h, protein expression of COX-2 was (51.8 ± 6.8)% and (34.9 ± 2.4)%, respectively (*P *< 0.05), and that of Survivin was (59.4 ± 6.9)% and (51.9 ± 5.6)% (*P *< 0.05).

### The alteration of apoptosis related factors after nimesulide treatment

**Figure 6:**
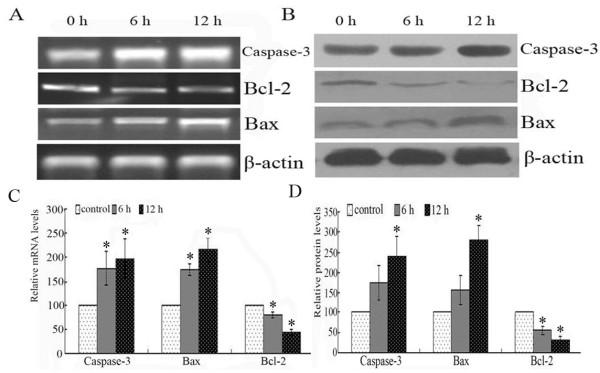
**The mRNA and protein levels of Bax, Bcl-2 and Caspase-3 in FaDu cells**. A: RT-PCR analysis. B: Western blot measurement. The optical densities (OD) value of mRNA (C) and proteins (D) in each lane using NIH software Image J system were demonstrated in different times treated with Nimesulide. Each data point in the figure represents the mean ± SEM of three separate experiments.

Nimesulide induced the expressions of Caspase-3 and Bax while Bcl-2 expression was down-regulated at both mRNA and protein levels (Figure 6). Taken the proteins expression control as 100%, Bcl-2 protein expression in the cells treated with Nimesulide for 6 h and 12 h were (55.1 ± 10.3)% and (32.1 ± 8.8)%, respectively (*P < 0.05*). The expression of Bax protein were increased to 1.56 ± 0.38 and 2.8 ± 0.35-folds (*P < 0.05*). The protein expression of Caspase-3 increased about 1.75 ± 0.44 and 2.41 ± 0.50-folds in the cells treated with Nimesulide for 6 h and 12 h, respectively (*P < 0.05*).

## Discussion

COX-2 is considered as an important potential regulator of cancer growth and progression. There are currently many lines of evidence from the literature that supports the use of COX-2 inhibitors for cancer chemoprevention in a variety of cancers [11]. However, relatively little research has investigated the anti-cancer effects of various COX-2 inhibitors on hypopharyngeal carcinoma. In this study, the viability of FaDu cells was suppressed by Nimesulide in time- and dose- dependent manners as evidenced by MTT assay, which was similar with the previous report that Nimesulide had an inhibitory effect of human ovarian SKOV-3 tumors [12]. This result could partly suggest that Nimesulide can reduce the growth of FaDu cells. We hypothesized this result may be based on two reasons. Firstly, the morphological test showed that FaDu cells, in response to Nimesulide, became rounded and had cytoplasmic contraction and chromatin condensation, which represents the typical morphological characteristics of apoptosis, and the Flow cytometry analysis demonstrated that the percentage of apoptosis were increased. Moreover, the BrdU positive cells detected in the control group were more than those in the treatment group. This indicated that Nimesulide was able to inhibit the proliferation of FaDu cells. Overall, our findings suggested that Nimesulide exerts its antitumor effect via anti-proliferation and apoptotic-inducing pathway.

Previous studies have demonstrated that down-regulation of COX-2 via siRNA treatment inhibited cell proliferation and induced apoptosis in human esophageal cancer cells [4]. As well known, the main role of selective COX-2 inhibitors is COX-2 activity suppress. The relationship between selective COX-2 inhibitor and COX-2 expression is controversial [13,14]. These contradictory results may be due to the different experiments systems (e.g. different drugs, dose and action time or different cell lines). To further understand the possible mechanism whereby Nimesulide-induced growth inhibition, we detected COX-2 expression in FaDu cells treated with Nimesulide. Similarly with previous reports, in the present study, we demonstrated a more significant reduction of COX-2 expression levels following the administration of Nimesulide in FaDu cells. Our data also supported the current concept that down-regulation of COX-2 may be involved in the regulation of Nimesulide-reduced growth.

As a regulator of apoptosis, Survivin play a very dominant effect in the anti-apoptotic process, and the over expression of Survivin induced unlimited proliferation of cancer cells. siRNA targeting Survivin inhibits growth and induces apoptosis in human renal clear cell carcinoma 786-O cells [15]. Previous studies have demonstrated that selective COX-2 inhibitors could induce apoptosis of cancer cells by inhibition of Survivin expression [16,17]. In present study, we found that Survivin expression both at the mRNA and protein levels were significantly reduced in the presence of Nimesulide in FaDu cells, as demonstrated by RT-PCR and Western blot analysis. The potent down-regulation of Survivin is reminiscent of the association between Survivin and the growth which was suppressed by Nimesulide.

To understand the potential mechanisms of Survivin-related in the process of Nimesulide-induced growth inhibition, we detected the downstream factors of it. In the present report, considering the well-known function of Survivin as an inhibitor of Caspases family, and Survivin knock down-induced apoptosis seemed to be Bcl-2 and Bax-dependent [18,19]. We detected the expression of Caspase-3, Bcl-2 and Bax in FaDu cells treated with Nimesulide. Our results demonstrated that Caspase-3 and Bax expression were potently up-regulated, and the expression of Bcl-2 was suppressed. All these results suggested that Survivin may play an important role in the Nimesulide-induced apoptosis.

In conclusion, Nimesulide could suppress the growth of hypopharyngeal carcinoma cells via anti-proliferation and apoptotic-inducing pathway in *vitro*, and the expression of Survivin may play an important role in the process. Our findings pave a way to selectively target COX-2 to inhibit the growth of hypopharyngeal carcinoma cells, and eventually for the treatment of hypopharyngeal carcinoma in the future.

## Competing interests

The authors declare that they have no competing interests.

## Authors' contributions

JJT and SML carried out the main research and analyzed data.LY, JKM and YKM helped in conducting the research. HBW and WX designed the research and wrote the paper. All authors read and approved the final manuscript.
